# Exclusive breastfeeding among women with type 1 and type 2 diabetes mellitus

**DOI:** 10.1186/s12884-022-04411-w

**Published:** 2022-01-27

**Authors:** Leandro Cordero, Michael R. Stenger, Mark B. Landon, Craig A. Nankervis

**Affiliations:** 1 Department of Pediatrics, College of Medicine, The Ohio State University Wexner Medical Center, N118 Doan Hall, 410 W. 10th Avenue, Columbus, OH 43210-1228 USA; 2 Department of Obstetrics, College of Medicine, The Ohio State University Wexner Medical Center, N118 Doan Hall, 410 W. 10th Avenue, Columbus, OH 43210-1228 USA

**Keywords:** Exclusive breastfeeding and type 1 and type 2 diabetes mellitus

## Abstract

**Objective:**

To compare exclusive breastfeeding (BF) and BF initiation among 185 women with Type 1 and 212 women with Type 2 pregestational diabetes who intended exclusive or partial BF and delivered at ≥34 weeks of gestation.

**Methods:**

Retrospective cohort study. At discharge, exclusive BF is direct BF or BF complemented with expressed breast milk. BF initiation is defined by exclusive or partial BF.

**Results:**

Type 1 and Type 2 groups were similar in prior BF experience (69 vs 67%) but were different in intention to BF exclusively (92 vs 78%) and partially (8 vs 22%). Women in the Type 1 group were younger (median age 30 vs 33y), likely to be primiparous (47 vs 25%), have a lower median BMI (32 vs 36 kg/m^2^) and deliver by primary cesarean (37 vs 26%). Infants born to Type 1 women were more likely to be admitted to the NICU (44 vs 18%) and to have hypoglycemia (59 vs 41%). At discharge, exclusive BF among Type 1 was higher (34 vs 23%), partial BF was similar (47 vs 46%) while FF (formula feeding) was lower (19 vs 31%) than in the Type 2 group. BF initiation occurred in 81% of Type 1 and 69% of Type 2 women.

**Conclusion:**

Intention to BF exclusively was higher in Type 1 women compared to Type 2. At discharge, exclusive BF and BF initiation were lower and FF higher in the Type 2 group highlighting the need for different strategies if lactation in this special population is to be improved.

## Background

Pregestational diabetes mellitus (Type 1 and Type 2) affects 1–1.5% of all pregnant women and may lead to adverse maternal and neonatal outcomes [1]. Other investigators noted an increase in pregestational diabetes as well as preeclampsia, chronic hypertension and obesity among women hospitalized for delivery in the US from 2005 through 2014 [2]. Additionally, investigators in California observed an alarming rise in the prevalence of Type 1 and Type 2 diabetes as well as racial/ethnic disparities in pregnant women from 1996 through 2014 [3]. Of the patients studied, 24% were classified as Type 1 and 76% as Type 2. Women in the Type 2 group tended to be older, African American or Hispanic, multiparous, had a high BMI and delivered by cesarean [3].

Earlier publications on breastfeeding (BF) focused mainly on women with Type 1 or reported small samples of Type 1 and Type 2 [4, 5]. A collaborative study confirmed that the duration of exclusive and overall BF was shorter in Type 1 than in non-diabetic women [6]. More recently, Herskin et al. compared 105 Type 1 with 44 Type 2 dyads and reported different BF rates at hospital discharge (76 vs 45%) and at 4 months postpartum (49 vs 23%) [7].

Lactation is critical because it provides short- and long-term health benefits to mothers and their infants following healthy pregnancies as well as those compromised by gestational and pregestational diabetes [8–11]. Despite the above, comparisons of exclusive BF and BF initiation rates among women with Type 1 and Type 2 diabetes remain scarce and deserves further study.

### Objective

To compare exclusive BF and BF initiation among 185 women with Type 1 and 212 women with Type 2 diabetes who intended exclusive or partial BF.

## Subjects and methods

This retrospective cohort investigation was approved by the Institutional Review Board at The Ohio State University Wexner Medical Center. Electronic maternal and neonatal records (2013–18) were reviewed. Type 1 and Type 2 pregestational diabetes, chronic hypertension (CHTN) and preeclampsia were diagnosed and treated in accordance with established guidelines [1, 12]. Women were categorized by prepregnancy BMI as normal (18–24.9 kg/m^2^), overweight (25–29.9 kg/m^2^), obese (30–39.9 kg/m^2^), morbidly obese (40–49.9 kg/m^2^) or extremely obese (≥ 50 kg/m^2^) [13].

All women delivered singleton infants at ≥34 weeks gestation and those affected by major fetal malformations were not included*.* Upon arrival to labor and delivery, each woman described her past BF experience and her intention to BF. In our institution, maternity practices include BF within 1 h, no formula supplementation unless indicated, rooming in, on demand BF, full-time lactation consultants and post discharge BF support [14–16]. Furthermore, our institution reports BF data to the Joint Commission as required for hospital accreditation [15].

Per our hospital practice, symptomatic infants were directly transferred from the delivery room to the NICU for further care. Following delivery if the condition of the mother and her infant allowed, maternal-infant interactions such as holding, skin-to-skin contact, and BF were encouraged. Asymptomatic infants able to feed were transferred to the newborn nursery for routine care and glucose monitoring [17].

Screening for hypoglycemia (blood glucose < 40 mg/dl) was done via serial point of care testing (Accu-Chek®) or by plasma glucose measurement in the laboratory (Beckman Coulter AU5800, Beckman Coulter Inc., Brea, CA, USA) starting within the first hour of life after the first feeding and every 2–4 h thereafter as needed. Asymptomatic infants in the newborn nursery with hypoglycemia were promptly BF or formula fed (FF) and those with recurrent hypoglycemia were transferred to the NICU for further care. On admission to the NICU, most infants were started on intravenous (IV) dextrose while those who were able to feed were BF or FF [17].

BF was considered early if given within the first two postpartum hours. Exclusive BF was defined as direct feedings from the breast, expressed breast milk (EBM) alone or in combination with direct BF or with donor human milk while hospitalized. Partial BF was defined as formula supplementation with direct BF or with EBM. BF was considered initiated if, during the 24 h preceding hospital discharge, infants were BF exclusively or BF partially [17]. Due to the retrospective study design, no follow-up information was available on infant feeding practices after hospital discharge.

## Statistical analysis

Comparisons between women with Type 1 and Type 2 pregestational diabetes were made with Mood’s median tests for continuous variables and Chi square tests for categorical variables. Significance was established at a *p* value < 0.05. To assess the normality of quantitative variables, we examined quantile-quantile (‘Q-Q’) plots for visible deviations from model assumptions. No such issues were seen. Univariate and multivariate stepwise logistic regression were used to ascertain the strength of association of Type 1 and Type 2 diabetes with exclusive BF and BF initiation at discharge controlling for maternal variables (CHTN, preeclampsia, mothers age, race, BMI, elevated hemoglobin A1c (HbA1c) levels (≥ 7.0%), multiparity, mode of delivery, prior BF, intention to BF exclusively, breastfed < 2 h from birth) and neonatal variables (gestational age, preterm, admission to NICU, neonatal hypoglycemia and infant length of stay). To test for collinearity between independent variables, we removed each one at a time from the full adjusted models presented and confirmed that the remaining estimates did not change drastically, which was always the case.

## Results

The study population consisted of 185 women with Type 1 and 212 women with Type 2 pregestational diabetes who intended to BF.

### Comparison of women with type 1 and type 2 diabetes

Clinical and demographic characteristics of women with Type 1 and Type 2 diabetes are shown in Table 1. The prevalence of CHTN alone (10 vs 18%) and preeclampsia superimposed on CHTN (8 vs 8%) were similar for Type 1 and Type 2 women. Preeclampsia as a single co-morbidity was more common among Type 1 than Type 2 women (21 vs 7%, *p* 0.0001). Preeclampsia alone or superimposed on CHTN combined affected 53 (29%) of Type 1 and 30 (14%) of Type 2 women. All women with preeclampsia with severe features received magnesium sulfate neuroprophylaxis for 24 h.

**Table 1:** Comparison of women with Type 1 and Type 2 Diabetes

	Type 1	Type 2	*p*
Mother-Infant dyads no.	185	212	
Pregestational DM without comorbidities no. (%)	114 (62)	144 (68)	NS
Chronic hypertension no. (%)	18 (10)	38 (18)	NS
Preeclampsia superimposed on CHTN no. (%)	15 (8)	16 (8)	NS
Preeclampsia no. (%)	38 (21)	14 (7)	0.0001
Mother’s age (y) median (IQR)	30 (26,34)	33 (29,36)	< 0.0001
Race			
African American no. (%)	40 (22)	52 (25)	NS
White no. (%)	133 (72)	73 (34)	0.0001
Hispanic no. (%)	7 (4)	55 (26)	0.0001
Other no. (%)	5 (3)	32 (15)	0.0001
Smoking during pregnancy no. (%)	10 (5)	16 (8)	NS
Former smokers no. (%)	22 (12)	34 (16)	NS
BMI kg/m^2^ median (IQR)	32 (28,37)	36 (31,42.5)	< 0.0001
Normal/overweight-BMI kg/m^2^ ≤ 29 no. (%)	64 (35)	28 (13)	0.0001
Obese-BMI kg/m^2^ 30–39 no. (%)	87 (47)	100 (47)	NS
Morbidly/extremely obese-BMI ≥ 40 no. (%)	34 (18)	84 (40)	0.0001
Multiparous no. (%)	98 (53)	159 (75)	0.0001
Mode of Delivery			
Vaginal no. (%)	67 (36)	85 (40)	NS
Primary cesarean no. (%)	68 (37)	56 (26)	0.03
Repeat cesarean no. (%)	50 (27)	71 (34)	NS
Mother length of stay (d) median (IQR)	4 (3,5)	4 (3,4)	0.002

Women with Type 2 diabetes tend to be older, multiparous, less often white, more often Hispanic and of other ethnicities. Median pregestational BMI was higher among African Americans (36.2 kg/m^2^) and Hispanics (35.0 kg/m^2^) than among whites (34.0 kg/m^2^) and women in the miscellaneous group (30.9 kg/m^2^). Normal/overweight (BMI ≤ 29 kg/m^2^) women were more common in Type 1 (35 vs 13%), obese women (BMI 30–39.9 kg/m^2^) were equally represented (47 vs 47%) and morbidly/extremely obese (≥ 40 kg/m^2^) women were more common in Type 2 (40 vs 18%). Single HbA1c determinations were recorded from all 185 Type 1 and from 98% of 212 Type 2 women. HbA1c tests were done after 20 weeks of gestation in 99% of the Type 1 and in 99% of the Type 2 women. Gestational age at the time of testing was a median 31 weeks (8-39w) for Type 1 and 32 weeks (15-39w) for Type 2 women. Elevated HbA1c (≥ 7.0%) was found in 43 of 185 Type 1 (23%) and in 39 of 207 (19%) of Type 2. Regression analysis showed that after adjusting for confounders, elevated HbA1c (≥ 7.0%) was not an independent predictor of exclusive BF or BF initiation for either Type 1 or Type 2 diabetic women.

### Neonatal outcomes of infants born to women with type 1 and type 2 diabetes

Infants born to Type 1 women were of lower median gestational age (37 vs 38w) and of higher median birthweight (3618 vs 3390 g) (Table 2). Both groups were similar in small for GA (3 vs 2%), large for GA (41 vs 37%) and appropriate for GA (57 vs 60%) infants. Of note, macrosomia (birthweight ≥4000 g) affected 23 and 22% of infants in Type 1 and Type 2 groups, respectively.

**Table 2:** Neonatal outcomes of infants born to women with Type 1 and Type 2 Diabetes

	Type 1	Type 2	*p*
Mother-Infant dyads no.	185	212	
Males no. (%)	94 (51)	114 (54)	NS
Birthweight (g) median (IQR)	3618 (3172, 3959)	3390 (3107,3922)	0.002
Gestational age (w) median (IQR)	37 (37,38)	38 (37,39)	0.02
Preterm no. (%)	40 (22)	34 (16)	NS
Intrauterine Growth			
Small for gestational age no. (%)	5 (3)	5 (2)	NS
Large for gestational age no. (%)	75 (41)	79 (37)	NS
Macrosomia no. (%)	43 (23)	47 (22)	NS
Admission to NICU no. (%)	82 (44)	38 (18)	0.0001
^a^Neonatal hypoglycemia no. (%)	109 (59)	86 (41)	0.0003
Infant length of stay (d) median (IQR)	3 (3,4)	3 (2.5,3.5)	0.08
Discharged home with mother no. (%)	173 (94)	200 (94)	NS

^a^Includes infants cared for in the Newborn Nursery or the NICU

Neonatal hypoglycemia and admission to the NICU occurred more often among infants in the Type 1 group than in the Type 2 group. Considering the similarities in diagnoses, 82 infants from the Type 1 group and 38 infants from the Type 2 group admitted to the NICU were combined for analysis. Seventy-five of the 120 (63%) infants were admitted to the NICU directly from the delivery room while the remaining 45 (37%) stayed in the newborn nursery for a short time prior to transfer. Primary admission diagnoses to the NICU included hypoglycemia (62%), respiratory distress (33%) and apnea-bradycardia-cyanosis (5%). Additionally, temperature instability-hypotonia-poor feeding affected one-third of all infants. Twenty-two of the 120 infants (18%) stayed in the NICU for less than 1 day, 23 infants (19%) stayed 2 days and the remaining 75 (63%) stayed 3 days or longer. All mothers and their infants were discharged home in good condition.

### Prior BF experience and early BF among women with type 1 and type 2 diabetes

The prevalence of multiparous women was lower in the Type 1 than in the Type 2 group (53 vs 75%), however, relevant to our study, prior BF experience among the multiparous women in either group was similar (69 vs 67%) (Table 3).

**Table 3:** Prior BF experience and early BF among women with Type 1 and Type 2 Diabetes

	Type 1	Type 2	*p*
Mother-Infant dyads no.	185	212	
Multiparous no. (%)	98 (53)	159 (75)	0.0001
Prior breastfeeding no. (%)	68 (69)	107 (67)	NS
Intended partial BF no. (%)	14 (8)	47 (22)	0.0001
Intended exclusive BF no. (%)	171 (92)	165 (78)	0.0001
Time to First Breastfeeding			
< 1 h no. (%)	74 (40)	87 (41)	NS
1–2 h no. (%)	8 (4)	19 (9)	NS
3–4 h no. (%)	19 (10)	15 (7)	NS
5–6 h no. (%)	11 (6)	10 (5)	NS
7–24 h no. (%)	32 (17)	32 (15)	NS
≥ 25 h no. (%)	20 (11)	18 (8)	NS
Never breastfed no. (%)	21 (12)	31 (15)	NS
Received lactation consult no. (%)	174 (94)	201 (95)	NS

Eighty-two of the 185 Type 1 (44%) and 106 of 212 (50%) of Type 2 infants BF during the first 2 h from birth.

Analysis of mode of delivery on early BF showed that of 67 women from the Type 1 group who delivered vaginally, 41 (61%) were able to BF within 2 h from birth, similar to those 57 of 85 (67%) in the Type 2 group. Among 118 women from the Type 1 group who delivered by cesarean, 39 (33%) were able to BF within 2 h from birth, similar to 49 of 127 (39%) of those in the Type 2 group.

### BF at discharge for women with type 1 and type 2 diabetes

At discharge, exclusive BF was 34% among Type 1 women and 23% for those in the Type 2 group (Table 4). In the Type 1 group were 171 women who intended exclusive BF, of them 61 (36%) BF exclusively at discharge. Conversely, of 14 others who intended partial BF only one (7%) BF exclusively at discharge. In the Type 2 group were 165 women who intended exclusive BF, of them 47 (28%) BF exclusively at discharge. In contrast, of 47 others who intended partial BF only 2 (4%) BF exclusively at discharge. Regression analysis showed that the stronger predictor of failure to exclusive BF at discharge for Type 1 women was neonatal hypoglycemia a OR 0.34 CI 95% (0.17, 0.68) and for Type 2 women were intention to partially BF a OR 0.10 CI 95% (0.02, 0.44) and neonatal hypoglycemia a OR 0.35 CI 95% (0.15, 0.79).

**Table 4:** BF at discharge for women with Type 1 and Type 2 Diabetes

	Type 1	Type 2	*p*
Mother-Infant dyads no.	185	212	
Multiparous no. (%)	98 (53)	159 (75)	0.0001
Prior Breastfeeding no. (%)	68 (69)	107 (67)	NS
Infant Feeding at Discharge			
Exclusive total no. (%)	62 (34)	49 (23)	0.02
Direct BF no. (%)	40 (65)	46 (94)	0.0001
Expressed breast milk/Donor no. (%)	22 (35)	3 (6)	0.0001
Partial total no. (%)	87 (47)	98 (46)	NS
Direct BF & Formula no. (%)	77 (89)	94 (96)	NS
Expressed breast milk & Formula no. (%)	10 (11)	4 (4)	NS
Formula feeding no. (%)	36 (19)	65 (31)	0.01
Breastfeeding initiation no. (%)	149 (81)	147 (69)	0.01

Of the 62 Type 1 infants in the exclusive BF subgroup, 40 received direct BF, 16 exclusive EBM and 6 exclusive donor human milk. Of the 49 infants in the Type 2 exclusive BF subgroup, 46 received direct BF, 2 exclusive EBM and 1 exclusive donor human milk. Partial BF rates were similar (47 vs 46%), formula feeding was lower (19 vs 31%) and BF initiation at discharge was higher (81 vs 69%) among Type 1 compared to Type 2 women. Regression analysis showed that the stronger predictors of BF initiation for Type 1 were BMI a OR 0.92 CI 95% (0.87, 0.99) and mothers age (a OR 1.07 CI 95% (0.99, 1.15). For infants in the Type 2 group the stronger predictors of BF initiation failure were intention to partially BF a OR 0.22 CI 95% (0.10, 0.50) and neonatal hypoglycemia a OR 0.35 CI 95% (0.15, 0.79).

Regardless of mode of delivery, 82 women from the Type 1 group (44%) and 106 women from the Type 2 group (50%) BF during the first 2 h. At discharge, 76 of 82 (93%) Type 1 and 91 of 106 (86%) Type 2, respectively, initiated BF. On the other hand, 73 of 103 (71%) women from the Type 1 and 49 of 106 (46%) women from the Type 2 group who did not BF within 2 h initiated BF at discharge.

A comparison of Type 1 and Type 2 women combined according to race, showed that BF initiation at discharge was observed in 85% of 206 whites, in 66% of 92 African Americans, in 64% of 62 Hispanics and in 81% of 37 women from the other group. Concurrently, the rate of exclusive BF was 29% for whites, 27% for the miscellaneous groups, 20% for African Americans and 19% for Hispanics. Logistic regression analysis showed that exclusive BF among Type 1 women was less likely among non-white women a OR 0.44 CI 95% (0.19, 1.00).

### Prior BF experience, exclusive BF and BF initiation in women with type 1 and type 2 diabetes

Inspite of the different prevalence of multiparous women, prior BF experience was similar among Type 1 and Type 2 groups. Further analysis showed that 68 Type 1 women with prior BF experience had higher exclusive BF (41 vs 29%) and BF initiation rates (88 vs 76%) than 117 others without prior BF experience. Among Type 2 women, 107 with prior experience had higher exclusive BF (27 vs 19%) and BF initiation rates (78 vs 61%) than 105 without prior BF experience. Logistic regression showed that prior BF was a strong predictor of exclusive BF among Type 1 a OR 4.07 CI 95% (1.21, 13.65) and among Type 2 a OR 3.96 CI 95% (1.82, 8.62). Similar analysis showed that prior BF was also a strong predictor of BF initiation among Type 1 a OR 4.12 CI 95% (1.10, 15.47) and among Type 2 women a OR 4.17 CI 95% (1.88, 9.28).

### Maternal obesity, type of diabetes and BF initiation

Sixty-four of 185 (35%) Type 1 and 28 of 212 (13%) Type 2 women were normal or overweight (BMI < 29 kg/m^2^). At discharge, exclusive BF (39 vs 36%) and BF initiation (87 vs 79%) were similar for both groups. Eighty-seven of 185 Type 1 (47%) and 100 of 212 (47%) of Type 2 women were obese (BMI 30–39.9 kg/m^2^). At discharge exclusive BF was higher (35 vs 22%, *p* 0.05) in the Type 1 group but BF initiation (80 vs 78%) was similar for both groups. Thirty-four of 185 (18%) Type 1 women and 84 of 212 (40%) Type 2 were morbidly/extremely obese (BMI ≥ 40 kg/m^2^). At discharge, exclusive BF (21 vs 20%) and BF initiation (71 vs 68%) were similar for both groups.

## Discussion

The American Academy of Pediatrics and the Academy of Breastfeeding Medicine recommend exclusive BF for all healthy infants during birth hospitalization and beyond [16, 18]. Since January 2014, the joint commission mandated exclusive BF during hospitalization for healthy infants to retain their maternity accreditation [15]. These organizations acknowledged that other nutritional options may be needed to temporarily replace or supplement BF under well-defined circumstances (i.e., maternal and neonatal illness, late preterm infants) [15, 18]. In a recent study of maternity care practices and policies in 1305 hospitals in the United States, the mean percentage of in-hospital exclusive BF infants in the general population was 51.4% [19]. Recently, we reported exclusive BF rates in women with mild (47%) and severe CHTN (50%) [20], with preeclampsia (39%) and with severe preeclampsia (37%) [21], with preeclampsia superimposed on pregestational diabetes (18%) [22], with CHTN superimposed on pregestational diabetes (19%) [23], with pregestational diabetes with (33%) and without prior BF experience (11%) [24]. The low exclusive BF rates for Type 1 (34%) and Type 2 (23%) women reported here are similar to those of women with pregestational diabetes with superimposed comorbidities described above [22–24].

In the US 83.2% of newborns initiated BF by the time of discharge from the birth hospital [25]. In the present study we observed a BF initiation rate of 81% for Type 1 and 69% for Type 2 women. Obstacles known to associate with low BF initiation affecting our study population included lack of prior BF experience, preeclampsia, CHTN, pregestational diabetes, obesity, complications of labor, cesarean delivery, premature birth, neonatal hypoglycemia, admission to NICU, late or absent BF, formula supplementation, delayed lactogenesis II and maternal infant separation [21, 26, 27]. Our regression analysis showed that the strong predictors of BF initiation for Type 1 were lower BMIs and younger maternal age, whereas among Type 2 predictors of BF initiation failure were intention to partially BF, higher BMI and neonatal hypoglycemia.

It is known that racial discrepancies in prevalence are accompanied by low rates of BF initiation and exclusive BF in African American and Hispanics as compared to non-Hispanic whites [28, 29]. In line with the above, our data on Type 1 and Type 2 combined also showed that exclusive BF and BF initiation among African American was lower than those of non-Hispanic white women.

Hypoglycemia, lack of early BF and admission to NICU are well known interrelated obstacles to exclusive BF and BF initiation among infants born to women with gestational and pregestational diabetes [17–26]. Timing of the first BF is important because many critical maternal and neonatal physiological interactions occur following delivery [17, 26, 30]. Persistent hypoglycemia is commonly found among infants born to women with Type 1 and Type 2 diabetes during the first hours after birth [17, 31]. Early BF or early FF may facilitate glycemic stability in infants born to women with pregestational diabetes and may prevent or correct hypoglycemia avoiding the need for dextrose gel, IV treatment or formula supplementation [17, 31].

Comorbidities like preeclampsia, CHTN, obesity, cesarean delivery, preterm birth, hypoglycemia, NICU admission and early BF may have all contributed to the low BF initiation observed in this investigation [20–23, 31, 32]. The high rate of cesarean deliveries in the Type 1 and Type 2 groups is not unexpected, especially for women with high-risk obstetrical conditions superimposed on obesity [27, 32, 33]. Women from either group who BF exclusively by EBM without any direct BF is also of concern because pumping without feeding at the breast is associated with shorter BF duration [34].

Prior BF experience is a predictor, albeit not absolute, of subsequent BF. In contrast, women who did not BF or who reported unsuccessful attempts to BF their first child are unlikely to BF subsequent children [35, 36]. At discharge, exclusive BF and BF initiation rates were higher for women with prior experience than for those without [24]. In the current study, Type 1 and Type 2 women with prior BF experience had better rates of exclusive BF and BF initiation than those without experience.

A positive BF experience improves attitude, confidence, self-efficacy, motivation and intention to BF [24, 35–37]. Negative BF experiences are related to maternal or neonatal morbidities or to difficulties inherent to lactation such as suck/latch problems, perception of low milk supply, mastitis or nipple fissures [24, 37]. Multiparous women who declare no prior BF experience represent a group that attempted to BF and were unsuccessful or who did not intend to BF [24, 35, 36]. A detailed BF history could provide insight into obstacles and may help to define appropriate preventive or corrective strategies for subsequent pregnancies [24].

The association of obesity, type of diabetes and BF initiation reported here is not surprising since obesity, especially if linked with delayed lactogenesis II, has a negative effect on rates of exclusive BF, BF initiation and BF duration [33]. Women who are obese are more likely to have comorbidities including preeclampsia, CHTN and preexisting diabetes and are at increased risk for cesarean delivery and fetal macrosomia, all factors known to negatively influence BF outcomes [20, 21, 33]. The lack of association of elevated HbA1c with exclusive BF or BF initiation in this study population was not unexpected because HbA1c level alone does not take into account glycemic variability especially during the third trimester [38, 39].

The major limitation of this investigation is that the definition of exclusive BF and BF initiation at discharge used here may be applicable only to women with high risk obstetrical conditions for whom early mother-infant interactions may be delayed [27]. Another limitation is the lack of follow-up information regarding infant feeding choices after discharge from the hospital. The strength of this investigation rests on the size of the obstetrical and neonatal population and the fact that the data were obtained directly from medical records and not from maternal recall or via post-delivery questionnaires [40].

In conclusion, intention to BF exclusively was higher in Type 1 women compared to Type 2. Exclusive BF in women with either Type 1 or Type 2 diabetes fell short of expectations, raising concerns about long term BF duration. Women and infants who are not ready for direct BF can be helped by temporary alternatives such as EBM or donor human milk. At discharge, exclusive BF and BF initiation were lower and FF higher in the Type 2 group, highlighting the need for different strategies if lactation in these special populations is to be improved.

## Data Availability

The data sets generated during and analyzed during the current study are not publicly available due to limitations of ethical approval involving the patient data and anonymity but are available from the corresponding author on reasonable request.
